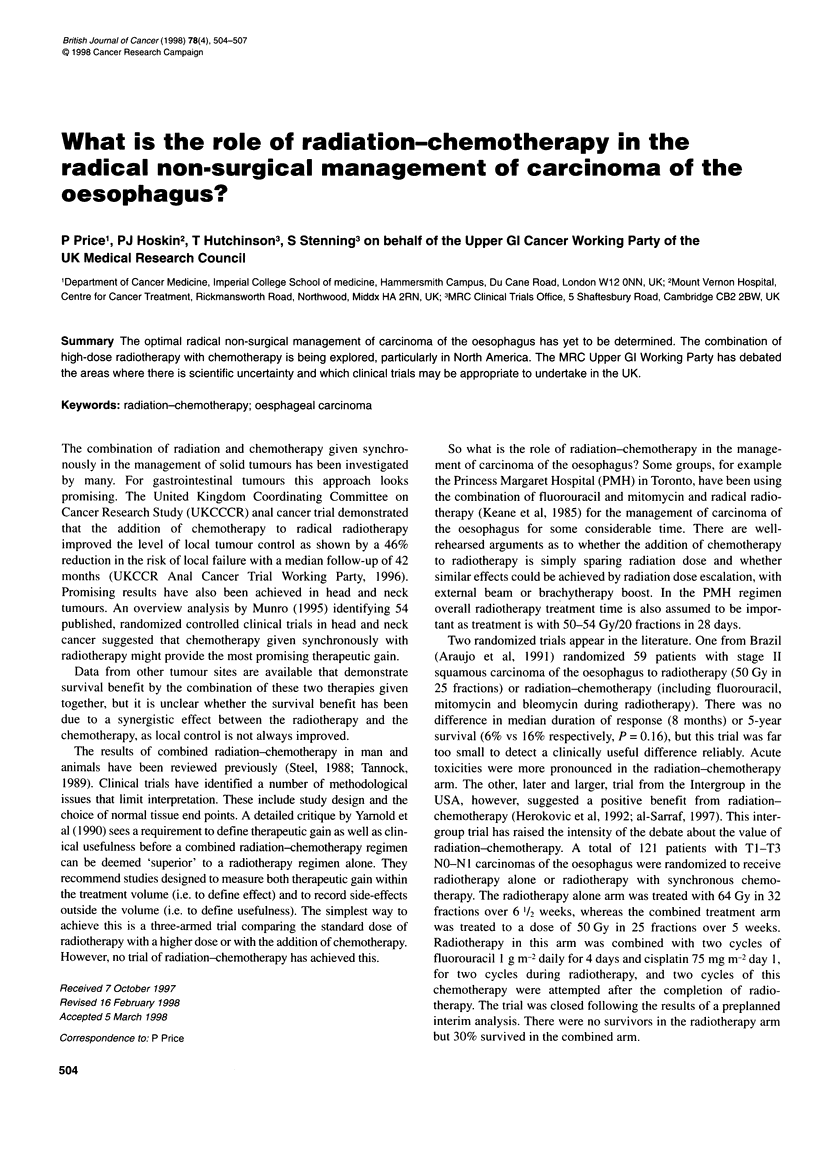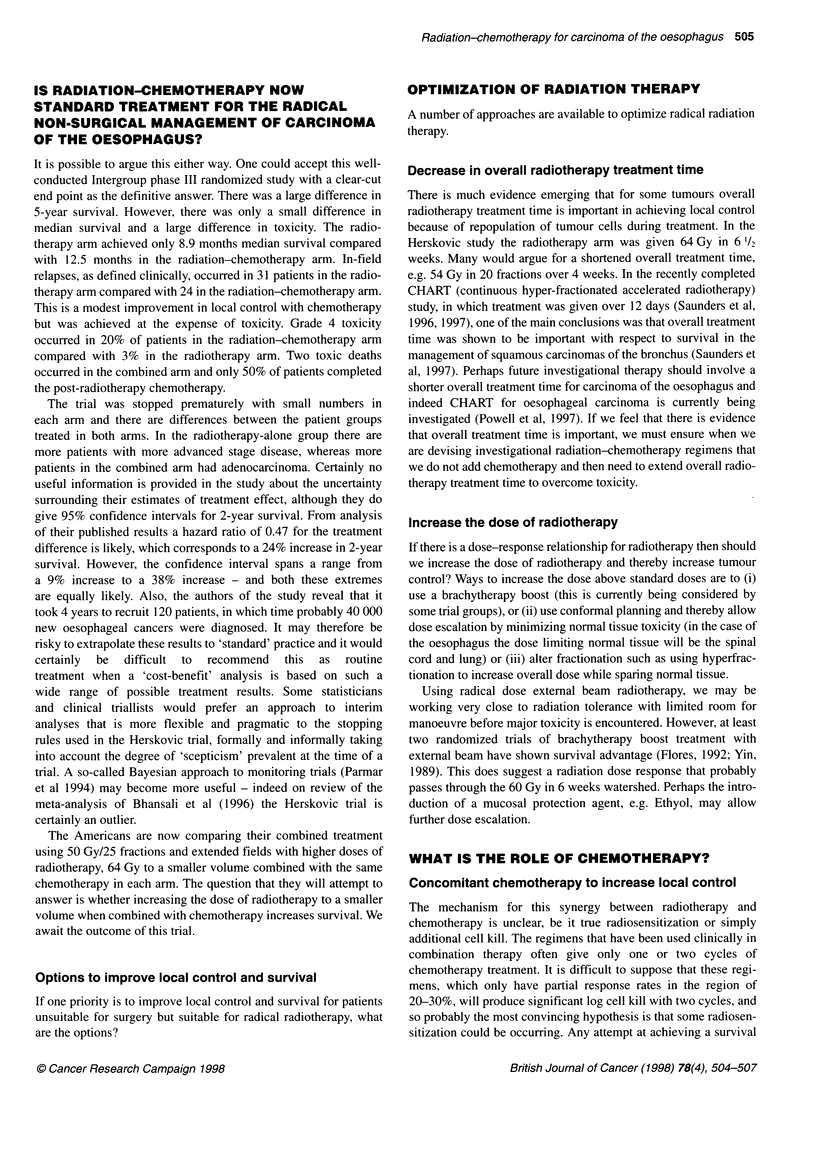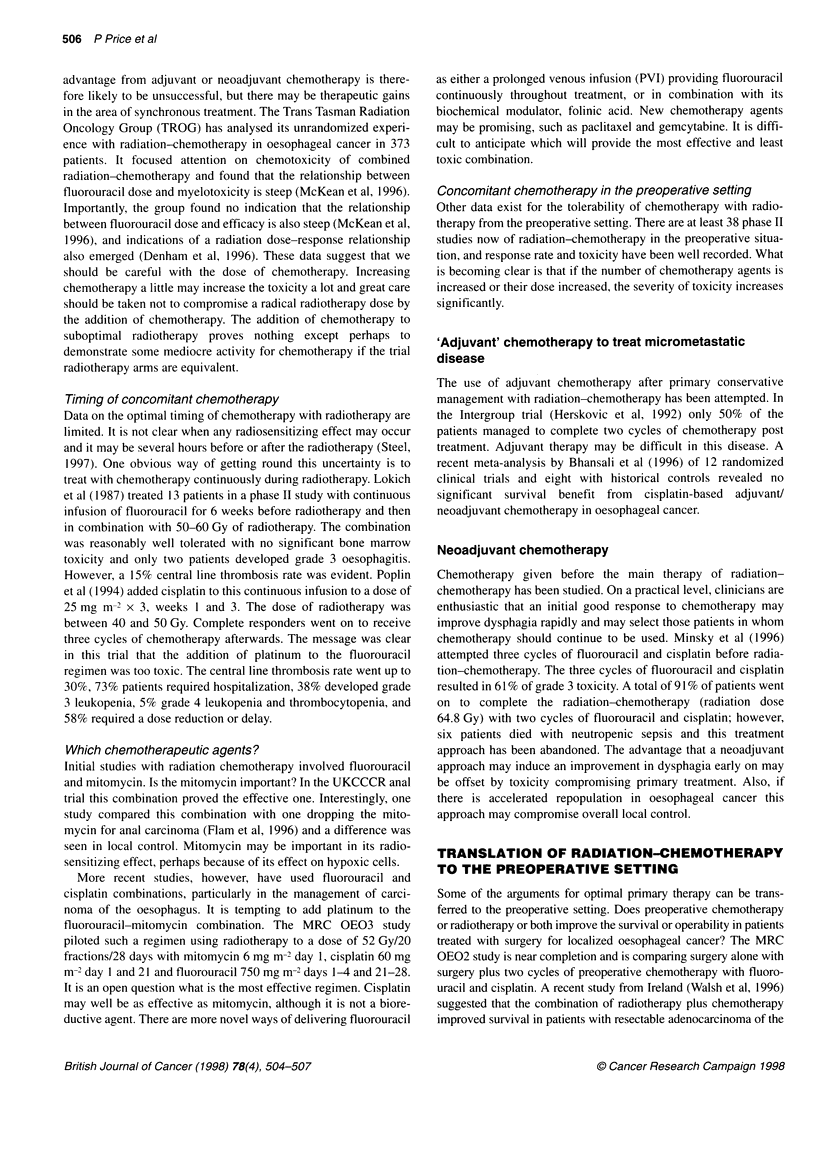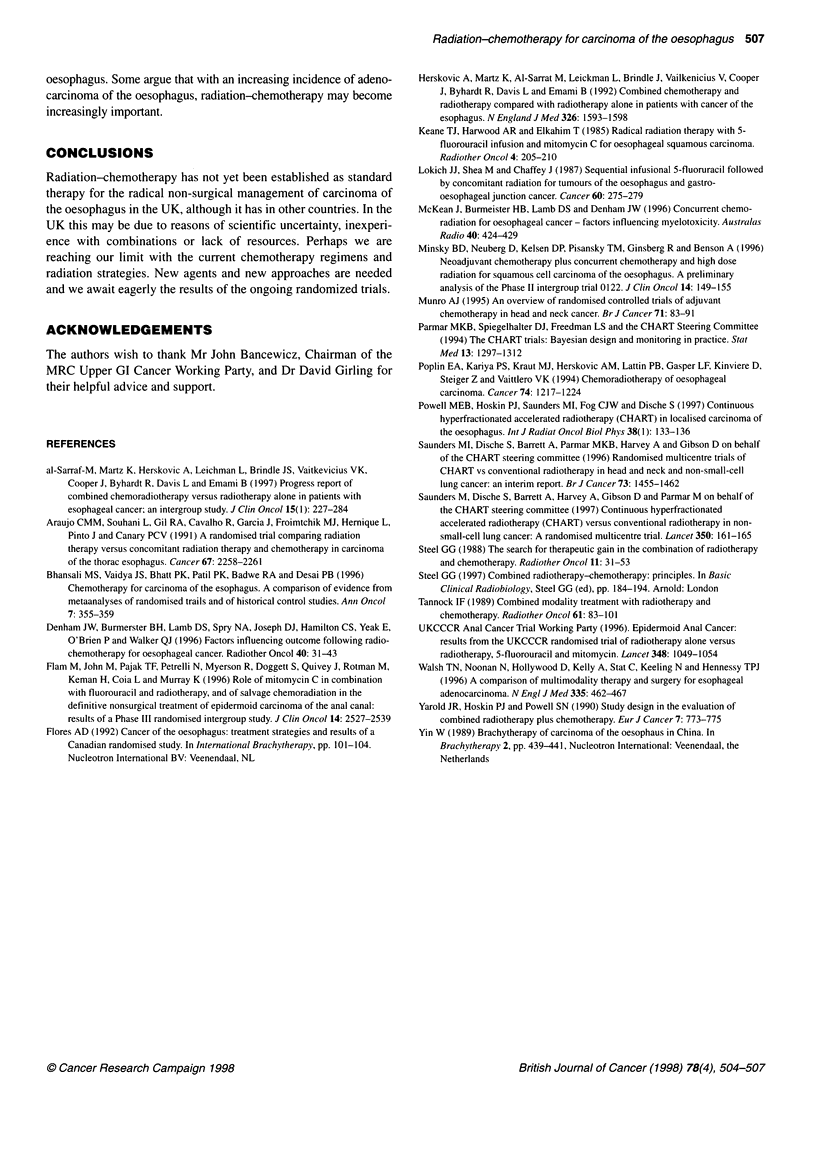# What is the role of radiation-chemotherapy in the radical non-surgical management of carcinoma of the oesophagus? Upper GI Cancer Working Party of the UK Medical Research Council.

**DOI:** 10.1038/bjc.1998.522

**Published:** 1998-08

**Authors:** P. Price, P. J. Hoskin, T. Hutchinson, S. Stenning

**Affiliations:** Department of Cancer Medicine, Imperial College School of Medicine, London, UK.

## Abstract

The optimal radical non-surgical management of carcinoma of the oesophagus has yet to be determined. The combination of high-dose radiotherapy with chemotherapy is being explored, particularly in North America. The MRC Upper GI Working Party has debated the areas where there is scientific uncertainty and which clinical trials may be appropriate to undertake in the UK.


British Journal of Cancer (1998) 78(4), 504–507
© 1998 Cancer Research Campaign

### What is the role of radiation–chemotherapy in the radical non-surgical management of carcinoma of the oesophagus?

**P Price[1], PJ Hoskin[2], T Hutchinson[3], S Stenning[3] on behalf of the Upper GI Cancer Working Party of the UK Medical Research Council**

[1]Department of Cancer Medicine, Imperial College School of medicine, Hammersmith Campus, Du Cane Road, London W12 0NN, UK; [2]Mount Vernon Hospital, Centre for Cancer Treatment, Rickmansworth Road, Northwood, Middx HA 2RN, UK; [3]MRC Clinical Trials Office, 5 Shaftesbury Road, Cambridge CB2 2BW, UK

**Summary** The optimal radical non-surgical management of carcinoma of the oesophagus has yet to be determined. The combination of high-dose radiotherapy with chemotherapy is being explored, particularly in North America. The MRC Upper GI Working Party has debated the areas where there is scientific uncertainty and which clinical trials may be appropriate to undertake in the UK.

**Keywords:** radiation–chemotherapy; oesphageal carcinoma

The combination of radiation and chemotherapy given synchronously in the management of solid tumours has been investigated by many. For gastrointestinal tumours this approach looks promising. The United Kingdom Coordinating Committee on Cancer Research Study (UKCCCR) anal cancer trial demonstrated that the addition of chemotherapy to radical radiotherapy improved the level of local tumour control as shown by a 46% reduction in the risk of local failure with a median follow-up of 42 months (UKCCR Anal Cancer Trial Working Party, 1996). Promising results have also been achieved in head and neck tumours. An overview analysis by Munro (1995) identifying 54 published, randomized controlled clinical trials in head and neck cancer suggested that chemotherapy given synchronously with radiotherapy might provide the most promising therapeutic gain.

Data from other tumour sites are available that demonstrate survival benefit by the combination of these two therapies given together, but it is unclear whether the survival benefit has been due to a synergistic effect between the radiotherapy and the chemotherapy, as local control is not always improved.

The results of combined radiation–chemotherapy in man and animals have been reviewed previously (Steel, 1988; Tannock, 1989). Clinical trials have identified a number of methodological issues that limit interpretation. These include study design and the choice of normal tissue end points. A detailed critique by Yarnold et al (1990) sees a requirement to define therapeutic gain as well as clinical usefulness before a combined radiation–chemotherapy regimen can be deemed 'superior' to a radiotherapy regimen alone. They recommend studies designed to measure both therapeutic gain within the treatment volume (i.e. to define effect) and to record side-effects outside the volume (i.e. to define usefulness). The simplest way to achieve this is a three-armed trial comparing the standard dose of radiotherapy with a higher dose or with the addition of chemotherapy. However, no trial of radiation–chemotherapy has achieved this.

*Received 7 October 1997*
*Revised 16 February 1998*
*Accepted 5 March 1998*

*Correspondence to:* P Price

So what is the role of radiation–chemotherapy in the management of carcinoma of the oesophagus? Some groups, for example the Princess Margaret Hospital (PMH) in Toronto, have been using the combination of fluorouracil and mitomycin and radical radiotherapy (Keane et al, 1985) for the management of carcinoma of the oesophagus for some considerable time. There are well-rehearsed arguments as to whether the addition of chemotherapy to radiotherapy is simply sparing radiation dose and whether similar effects could be achieved by radiation dose escalation, with external beam or brachytherapy boost. In the PMH regimen overall radiotherapy treatment time is also assumed to be important as treatment is with 50–54 Gy/20 fractions in 28 days.

Two randomized trials appear in the literature. One from Brazil (Araujo et al, 1991) randomized 59 patients with stage II squamous carcinoma of the oesophagus to radiotherapy (50 Gy in 25 fractions) or radiation–chemotherapy (including fluorouracil, mitomycin and bleomycin during radiotherapy). There was no difference in median duration of response (8 months) or 5-year survival (6% vs 16% respectively, $P = 0.16$), but this trial was far too small to detect a clinically useful difference reliably. Acute toxicities were more pronounced in the radiation–chemotherapy arm. The other, later and larger, trial from the Intergroup in the USA, however, suggested a positive benefit from radiation–chemotherapy (Herokovic et al, 1992; al-Sarraf, 1997). This intergroup trial has raised the intensity of the debate about the value of radiation–chemotherapy. A total of 121 patients with T1–T3 N0–N1 carcinomas of the oesophagus were randomized to receive radiotherapy alone or radiotherapy with synchronous chemotherapy. The radiotherapy alone arm was treated with 64 Gy in 32 fractions over 6 ½ weeks, whereas the combined treatment arm was treated to a dose of 50 Gy in 25 fractions over 5 weeks. Radiotherapy in this arm was combined with two cycles of fluorouracil 1 g $m^{-2}$ daily for 4 days and cisplatin 75 mg $m^{-2}$ day 1, for two cycles during radiotherapy, and two cycles of this chemotherapy were attempted after the completion of radiotherapy. The trial was closed following the results of a preplanned interim analysis. There were no survivors in the radiotherapy arm but 30% survived in the combined arm.

### IS RADIATION–CHEMOTHERAPY NOW STANDARD TREATMENT FOR THE RADICAL NON-SURGICAL MANAGEMENT OF CARCINOMA OF THE OESOPHAGUS?

It is possible to argue this either way. One could accept this well-conducted Intergroup phase III randomized study with a clear-cut end point as the definitive answer. There was a large difference in 5-year survival. However, there was only a small difference in median survival and a large difference in toxicity. The radiotherapy arm achieved only 8.9 months median survival compared with 12.5 months in the radiation–chemotherapy arm. In-field relapses, as defined clinically, occurred in 31 patients in the radiotherapy arm compared with 24 in the radiation–chemotherapy arm. This is a modest improvement in local control with chemotherapy but was achieved at the expense of toxicity. Grade 4 toxicity occurred in 20% of patients in the radiation–chemotherapy arm compared with 3% in the radiotherapy arm. Two toxic deaths occurred in the combined arm and only 50% of patients completed the post-radiotherapy chemotherapy.

The trial was stopped prematurely with small numbers in each arm and there are differences between the patient groups treated in both arms. In the radiotherapy-alone group there are more patients with more advanced stage disease, whereas more patients in the combined arm had adenocarcinoma. Certainly no useful information is provided in the study about the uncertainty surrounding their estimates of treatment effect, although they do give 95% confidence intervals for 2-year survival. From analysis of their published results a hazard ratio of 0.47 for the treatment difference is likely, which corresponds to a 24% increase in 2-year survival. However, the confidence interval spans a range from a 9% increase to a 38% increase – and both these extremes are equally likely. Also, the authors of the study reveal that it took 4 years to recruit 120 patients, in which time probably 40 000 new oesophageal cancers were diagnosed. It may therefore be risky to extrapolate these results to 'standard' practice and it would certainly be difficult to recommend this as routine treatment when a 'cost-benefit' analysis is based on such a wide range of possible treatment results. Some statisticians and clinical triallists would prefer an approach to interim analyses that is more flexible and pragmatic to the stopping rules used in the Herskovic trial, formally and informally taking into account the degree of 'scepticism' prevalent at the time of a trial. A so-called Bayesian approach to monitoring trials (Parmar et al 1994) may become more useful – indeed on review of the meta-analysis of Bhansali et al (1996) the Herskovic trial is certainly an outlier.

The Americans are now comparing their combined treatment using 50 Gy/25 fractions and extended fields with higher doses of radiotherapy, 64 Gy to a smaller volume combined with the same chemotherapy in each arm. The question that they will attempt to answer is whether increasing the dose of radiotherapy to a smaller volume when combined with chemotherapy increases survival. We await the outcome of this trial.

### Options to improve local control and survival

If one priority is to improve local control and survival for patients unsuitable for surgery but suitable for radical radiotherapy, what are the options?

### OPTIMIZATION OF RADIATION THERAPY

A number of approaches are available to optimize radical radiation therapy.

### Decrease in overall radiotherapy treatment time

There is much evidence emerging that for some tumours overall radiotherapy treatment time is important in achieving local control because of repopulation of tumour cells during treatment. In the Herskovic study the radiotherapy arm was given 64 Gy in 6 $^1/_2$ weeks. Many would argue for a shortened overall treatment time, e.g. 54 Gy in 20 fractions over 4 weeks. In the recently completed CHART (continuous hyper-fractionated accelerated radiotherapy) study, in which treatment was given over 12 days (Saunders et al, 1996, 1997), one of the main conclusions was that overall treatment time was shown to be important with respect to survival in the management of squamous carcinomas of the bronchus (Saunders et al, 1997). Perhaps future investigational therapy should involve a shorter overall treatment time for carcinoma of the oesophagus and indeed CHART for oesophageal carcinoma is currently being investigated (Powell et al, 1997). If we feel that there is evidence that overall treatment time is important, we must ensure when we are devising investigational radiation–chemotherapy regimens that we do not add chemotherapy and then need to extend overall radiotherapy treatment time to overcome toxicity.

### Increase the dose of radiotherapy

If there is a dose–response relationship for radiotherapy then should we increase the dose of radiotherapy and thereby increase tumour control? Ways to increase the dose above standard doses are to (i) use a brachytherapy boost (this is currently being considered by some trial groups), or (ii) use conformal planning and thereby allow dose escalation by minimizing normal tissue toxicity (in the case of the oesophagus the dose limiting normal tissue will be the spinal cord and lung) or (iii) alter fractionation such as using hyperfractionation to increase overall dose while sparing normal tissue.

Using radical dose external beam radiotherapy, we may be working very close to radiation tolerance with limited room for manoeuvre before major toxicity is encountered. However, at least two randomized trials of brachytherapy boost treatment with external beam have shown survival advantage (Flores, 1992; Yin, 1989). This does suggest a radiation dose response that probably passes through the 60 Gy in 6 weeks watershed. Perhaps the introduction of a mucosal protection agent, e.g. Ethyol, may allow further dose escalation.

### WHAT IS THE ROLE OF CHEMOTHERAPY?

### Concomitant chemotherapy to increase local control

The mechanism for this synergy between radiotherapy and chemotherapy is unclear, be it true radiosensitization or simply additional cell kill. The regimens that have been used clinically in combination therapy often give only one or two cycles of chemotherapy treatment. It is difficult to suppose that these regimens, which only have partial response rates in the region of 20–30%, will produce significant log cell kill with two cycles, and so probably the most convincing hypothesis is that some radiosensitization could be occurring. Any attempt at achieving a survival

© Cancer Research Campaign 1998

advantage from adjuvant or neoadjuvant chemotherapy is therefore likely to be unsuccessful, but there may be therapeutic gains in the area of synchronous treatment. The Trans Tasman Radiation Oncology Group (TROG) has analysed its unrandomized experience with radiation–chemotherapy in oesophageal cancer in 373 patients. It focused attention on chemotoxicity of combined radiation–chemotherapy and found that the relationship between fluorouracil dose and myelotoxicity is steep (McKean et al, 1996). Importantly, the group found no indication that the relationship between fluorouracil dose and efficacy is also steep (McKean et al, 1996), and indications of a radiation dose–response relationship also emerged (Denham et al, 1996). These data suggest that we should be careful with the dose of chemotherapy. Increasing chemotherapy a little may increase the toxicity a lot and great care should be taken not to compromise a radical radiotherapy dose by the addition of chemotherapy. The addition of chemotherapy to suboptimal radiotherapy proves nothing except perhaps to demonstrate some mediocre activity for chemotherapy if the trial radiotherapy arms are equivalent.

### *Timing of concomitant chemotherapy*

Data on the optimal timing of chemotherapy with radiotherapy are limited. It is not clear when any radiosensitizing effect may occur and it may be several hours before or after the radiotherapy (Steel, 1997). One obvious way of getting round this uncertainty is to treat with chemotherapy continuously during radiotherapy. Lokich et al (1987) treated 13 patients in a phase II study with continuous infusion of fluorouracil for 6 weeks before radiotherapy and then in combination with 50–60 Gy of radiotherapy. The combination was reasonably well tolerated with no significant bone marrow toxicity and only two patients developed grade 3 oesophagitis. However, a 15% central line thrombosis rate was evident. Poplin et al (1994) added cisplatin to this continuous infusion to a dose of 25 mg $m^{-2}$ × 3, weeks 1 and 3. The dose of radiotherapy was between 40 and 50 Gy. Complete responders went on to receive three cycles of chemotherapy afterwards. The message was clear in this trial that the addition of platinum to the fluorouracil regimen was too toxic. The central line thrombosis rate went up to 30%, 73% patients required hospitalization, 38% developed grade 3 leukopenia, 5% grade 4 leukopenia and thrombocytopenia, and 58% required a dose reduction or delay.

### *Which chemotherapeutic agents?*

Initial studies with radiation chemotherapy involved fluorouracil and mitomycin. Is the mitomycin important? In the UKCCCR anal trial this combination proved the effective one. Interestingly, one study compared this combination with one dropping the mitomycin for anal carcinoma (Flam et al, 1996) and a difference was seen in local control. Mitomycin may be important in its radiosensitizing effect, perhaps because of its effect on hypoxic cells.

More recent studies, however, have used fluorouracil and cisplatin combinations, particularly in the management of carcinoma of the oesophagus. It is tempting to add platinum to the fluorouracil–mitomycin combination. The MRC OEO3 study piloted such a regimen using radiotherapy to a dose of 52 Gy/20 fractions/28 days with mitomycin 6 mg $m^{-2}$ day 1, cisplatin 60 mg $m^{-2}$ day 1 and 21 and fluorouracil 750 mg $m^{-2}$ days 1–4 and 21–28. It is an open question what is the most effective regimen. Cisplatin may well be as effective as mitomycin, although it is not a bioreductive agent. There are more novel ways of delivering fluorouracil as either a prolonged venous infusion (PVI) providing fluorouracil continuously throughout treatment, or in combination with its biochemical modulator, folinic acid. New chemotherapy agents may be promising, such as paclitaxel and gemcytabine. It is difficult to anticipate which will provide the most effective and least toxic combination.

### *Concomitant chemotherapy in the preoperative setting*

Other data exist for the tolerability of chemotherapy with radiotherapy from the preoperative setting. There are at least 38 phase II studies now of radiation–chemotherapy in the preoperative situation, and response rate and toxicity have been well recorded. What is becoming clear is that if the number of chemotherapy agents is increased or their dose increased, the severity of toxicity increases significantly.

### 'Adjuvant' chemotherapy to treat micrometastatic disease

The use of adjuvant chemotherapy after primary conservative management with radiation–chemotherapy has been attempted. In the Intergroup trial (Herskovic et al, 1992) only 50% of the patients managed to complete two cycles of chemotherapy post treatment. Adjuvant therapy may be difficult in this disease. A recent meta-analysis by Bhansali et al (1996) of 12 randomized clinical trials and eight with historical controls revealed no significant survival benefit from cisplatin-based adjuvant/neoadjuvant chemotherapy in oesophageal cancer.

### Neoadjuvant chemotherapy

Chemotherapy given before the main therapy of radiation–chemotherapy has been studied. On a practical level, clinicians are enthusiastic that an initial good response to chemotherapy may improve dysphagia rapidly and may select those patients in whom chemotherapy should continue to be used. Minsky et al (1996) attempted three cycles of fluorouracil and cisplatin before radiation–chemotherapy. The three cycles of fluorouracil and cisplatin resulted in 61% of grade 3 toxicity. A total of 91% of patients went on to complete the radiation–chemotherapy (radiation dose 64.8 Gy) with two cycles of fluorouracil and cisplatin; however, six patients died with neutropenic sepsis and this treatment approach has been abandoned. The advantage that a neoadjuvant approach may induce an improvement in dysphagia early on may be offset by toxicity compromising primary treatment. Also, if there is accelerated repopulation in oesophageal cancer this approach may compromise overall local control.

### TRANSLATION OF RADIATION–CHEMOTHERAPY TO THE PREOPERATIVE SETTING

Some of the arguments for optimal primary therapy can be transferred to the preoperative setting. Does preoperative chemotherapy or radiotherapy or both improve the survival or operability in patients treated with surgery for localized oesophageal cancer? The MRC OEO2 study is near completion and is comparing surgery alone with surgery plus two cycles of preoperative chemotherapy with fluorouracil and cisplatin. A recent study from Ireland (Walsh et al, 1996) suggested that the combination of radiotherapy plus chemotherapy improved survival in patients with resectable adenocarcinoma of the

© Cancer Research Campaign 1998

oesophagus. Some argue that with an increasing incidence of adenocarcinoma of the oesophagus, radiation–chemotherapy may become increasingly important.

### CONCLUSIONS

Radiation–chemotherapy has not yet been established as standard therapy for the radical non-surgical management of carcinoma of the oesophagus in the UK, although it has in other countries. In the UK this may be due to reasons of scientific uncertainty, inexperience with combinations or lack of resources. Perhaps we are reaching our limit with the current chemotherapy regimens and radiation strategies. New agents and new approaches are needed and we await eagerly the results of the ongoing randomized trials.

### ACKNOWLEDGEMENTS

The authors wish to thank Mr John Bancewicz, Chairman of the MRC Upper GI Cancer Working Party, and Dr David Girling for their helpful advice and support.

### REFERENCES

al-Sarraf-M, Martz K, Herskovic A, Leichman L, Brindle JS, Vaitkevicius VK, Cooper J, Byhardt R, Davis L and Emami B (1997) Progress report of combined chemoradiotherapy versus radiotherapy alone in patients with esophageal cancer; an intergroup study. *J Clin Oncol* **15**(1): 227–284

Araujo CMM, Souhani L, Gil RA, Cavalho R, Garcia J, Froimtchik MJ, Hernique L, Pinto J and Canary PCV (1991) A randomised trial comparing radiation therapy versus concomitant radiation therapy and chemotherapy in carcinoma of the thorac esophagus. *Cancer* **67**: 2258–2261

Bhansali MS, Vaidya JS, Bhatt PK, Patil PK, Badwe RA and Desai PB (1996) Chemotherapy for carcinoma of the esophagus. A comparison of evidence from metaanalyses of randomised trails and of historical control studies. *Ann Oncol* **7**: 355–359

Denham JW, Burmerster BH, Lamb DS, Spry NA, Joseph DJ, Hamilton CS, Yeak E, O'Brien P and Walker QJ (1996) Factors influencing outcome following radiochemotherapy for oesophageal cancer. Radiother Oncol **40**: 31–43

Flam M, John M, Pajak TF, Petrelli N, Myerson R, Doggett S, Quivey J, Rotman M, Keman H, Coia L and Murray K (1996) Role of mitomycin C in combination with fluorouracil and radiotherapy, and of salvage chemoradiation in the definitive nonsurgical treatment of epidermoid carcinoma of the anal canal: results of a Phase III randomised intergroup study. *J Clin Oncol* **14**: 2527–2539

Flores AD (1992) Cancer of the oesophagus: treatment strategies and results of a Canadian randomised study. In *International Brachytherapy*, pp. 101–104. Nucleotron International BV: Veenendaal, NL

Herskovic A, Martz K, Al-Sarrat M, Leickman L, Brindle J, Vailkenicius V, Cooper J, Byhardt R, Davis L and Emami B (1992) Combined chemotherapy and radiotherapy compared with radiotherapy alone in patients with cancer of the esophagus. *N England J Med* **326**: 1593–1598

Keane TJ, Harwood AR and Elkahim T (1985) Radical radiation therapy with 5-fluorouracil infusion and mitomycin C for oesophageal squamous carcinoma. *Radiother Oncol* **4**: 205–210

Lokich JJ, Shea M and Chaffey J (1987) Sequential infusional 5-fluoruracil followed by concomitant radiation for tumours of the oesophagus and gastro-oesophageal junction cancer. *Cancer* **60**: 275–279

McKean J, Burmeister HB, Lamb DS and Denham JW (1996) Concurrent chemo-radiation for oesophageal cancer – factors influencing myelotoxicity. *Australas Radio* **40**: 424–429

Minsky BD, Neuberg D, Kelsen DP, Pisansky TM, Ginsberg R and Benson A (1996) Neoadjuvant chemotherapy plus concurrent chemotherapy and high dose radiation for squamous cell carcinoma of the oesophagus. A preliminary analysis of the Phase II intergroup trial 0122. *J Clin Oncol* **14**: 149–155

Munro AJ (1995) An overview of randomised controlled trials of adjuvant chemotherapy in head and neck cancer. *Br J Cancer* **71**: 83–91

Parmar MKB, Spiegelhalter DJ, Freedman LS and the CHART Steering Committee (1994) The CHART trials: Bayesian design and monitoring in practice. *Stat Med* **13**: 1297–1312

Poplin EA, Kariya PS, Kraut MJ, Herskovic AM, Lattin PB, Gasper LF, Kinviere D, Steiger Z and Vaittlero VK (1994) Chemoradiotherapy of oesophageal carcinoma. *Cancer* **74**: 1217–1224

Powell MEB, Hoskin PJ, Saunders MI, Fog CJW and Dische S (1997) Continuous hyperfractionated accelerated radiotherapy (CHART) in localised carcinoma of the oesophagus. *Int J Radiat Oncol Biol Phys* **38**(1): 133–136

Saunders MI, Dische S, Barrett A, Parmar MKB, Harvey A and Gibson D on behalf of the CHART steering committee (1996) Randomised multicentre trials of CHART vs conventional radiotherapy in head and neck and non-small-cell lung cancer: an interim report. *Br J Cancer* **73**: 1455–1462

Saunders M, Dische S, Barrett A, Harvey A, Gibson D and Parmar M on behalf of the CHART steering committee (1997) Continuous hyperfractionated accelerated radiotherapy (CHART) versus conventional radiotherapy in non-small-cell lung cancer: A randomised multicentre trial. *Lancet* **350**: 161–165

Steel GG (1988) The search for therapeutic gain in the combination of radiotherapy and chemotherapy. *Radiother Oncol* **11**: 31–53

Steel GG (1997) Combined radiotherapy–chemotherapy: principles. In *Basic Clinical Radiobiology*, Steel GG (ed), pp. 184–194. Arnold: London

Tannock IF (1989) Combined modality treatment with radiotherapy and chemotherapy. *Radiother Oncol* **61**: 83–101

UKCCCR Anal Cancer Trial Working Party (1996). Epidermoid Anal Cancer: results from the UKCCCR randomised trial of radiotherapy alone versus radiotherapy, 5-fluorouracil and mitomycin. *Lancet* **348**: 1049–1054

Walsh TN, Noonan N, Hollywood D, Kelly A, Stat C, Keeling N and Hennessy TPJ (1996) A comparison of multimodality therapy and surgery for esophageal adenocarcinoma. *N Engl J Med* **335**: 462–467

Yarold JR, Hoskin PJ and Powell SN (1990) Study design in the evaluation of combined radiotherapy plus chemotherapy. *Eur J Cancer* **7**: 773–775

Yin W (1989) Brachytherapy of carcinoma of the oesophaus in China. In *Brachytherapy* **2**, pp. 439–441, Nucleotron International: Veenendaal, the Netherlands

© Cancer Research Campaign 1998